# Quantification of Rhodojaponin II and Rhodojaponin III in Rat Plasma by Ultra-Performance Liquid Chromatography-Tandem Mass Spectrometry

**DOI:** 10.1155/2024/6386570

**Published:** 2024-06-18

**Authors:** Cheng Sun, Wanhang Wang, Xi Bao, Dizhong Chen, Shenshen Mei, Jianshe Ma, Xiajuan Jiang

**Affiliations:** ^1^Department of Pharmacy, The Affiliated Hospital of Hangzhou Normal University, Hangzhou, China; ^2^Laboratory Animal Centre, Wenzhou Medical University, Wenzhou, China; ^3^Department of Pharmacy, The First Affiliated Hospital of Wenzhou Medical University, Wenzhou, China; ^4^School of Basic Medical Sciences, Wenzhou Medical University, Wenzhou, China

## Abstract

An ultra-performance liquid chromatography-tandem mass spectrometry (UPLC-MS/MS) method was developed to determine the concentrations of Rhodojaponin II and Rhodojaponin III in rat plasma, and their pharmacokinetic profiles were investigated. A UPLC HSS T3 (2.1 mm × 50 mm, 1.8 *μ*m) chromatographic column was employed at a temperature of 40°C. The mobile phase consisted of acetonitrile-0.1% formic acid in water, and a gradient elution method with an elution time of 6 min and flow rate of 0.4 mL/min was utilized for analysis purposes. Methodological investigations were conducted accordingly. The plasma concentrations of Rhodojaponin II and Rhodojaponin III exhibited excellent linearity within the range of 2 ng/mL–1250 ng/mL. Moreover, both intraday and interday precision were below 15%, while accuracy ranged from 88% to 115%. Additionally, matrix effect fell within the range of 90%–110%, and recoveries ranged from 78% to 87%. These results comply with relevant regulations for drug analysis in biological samples. Therefore, this method is deemed suitable for quantifying Rhodojaponin II and Rhodojaponin III levels in rats.

## 1. Introduction

*Rhododendron molle* (Blum) G. Don, a species of shrub, has gained popularity as an ornamental plant in recent years and is widely recognized by the public [1]. However, its medicinal properties derived from the roots, flowers, and fruits remain relatively unknown. Extensive research has demonstrated that each medicinal component of this plant exhibits diverse effects such as anti-inflammatory and analgesic properties, antipyretic activity, and hypotensive effects [2]. Despite its therapeutic potential, it should be noted that *Rhododendron molle* possesses significant toxicity with no specific treatment available; hence, clinical management primarily focuses on symptomatic relief [3]. Toxic symptoms include nausea, vomiting, diarrhea, bradycardia, hypotension, dyskinesia, and dyspnea which can lead to fatal outcomes in severe cases [1]. Consequently, the flowers are categorized as toxic herbs requiring careful handling. Studies have identified diterpenoids like Rhodojaponin II and Rhodojaponin III as the primary active and toxic constituents of *Rhododendron molle* (Blum) G. Don [4–6]. Diterpenoids exhibit potent insecticidal properties while also reducing blood pressure and heart rate along with inhibiting inflammatory responses. Additionally, it demonstrates remarkable analgesic efficacy. Notably, Rhodojaponin III surpasses morphine in terms of acute pain models as well as inflammatory pain models. Its potency for diabetic neuropathic pain models is 100 times greater than gabapentin's effectiveness [2, 7]. However, it was discovered that both intraperitoneal administration and oral consumption of Rhodojaponin III resulted in severe acute toxicity in mice. Moreover, it was implicated as one of the components associated with cardiotoxicity observed in rats treated with orally administered *Rhododendri Mollis* Flos extract. To date, research on Rhodojaponin II and Rhodojaponin III remains limited.

The present study establishes an analytical method based on ultra-performance liquid chromatography-tandem mass spectrometry (UPLC-MS/MS) and successfully applies it to determine the concentrations of Rhodojaponin II and Rhodojaponin III in rat plasma. This approach aims to elucidate the metabolic characteristics of these compounds in rats, providing valuable insights for their rational use in clinical practice.

## 2. Experimental

### 2.1. Reagents

**Figure 1:**
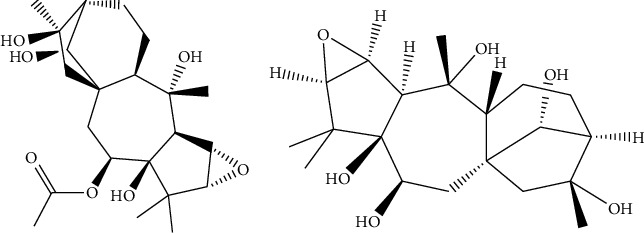
Chemical structures of Rhodojaponin II (a) and Rhodojaponin III (b).

Chromatographically pure acetonitrile and methanol were procured from Merck GmbH (Darmstadt, Germany). Chromatographically pure formic acid was obtained from Tedia Ltd. (Ohio, USA). Rhodojaponin II and Rhodojaponin III (purity >98%, Figure 1) were sourced from Chengdu Manster Biotechnology Co. (Chengdu, China). Ultrapure water was generated using the Millipore Milli-Q purification system (Bedford, MA, USA).

### 2.2. Animals

The healthy male Sprague–Dawley (SD) rats weighing 200 g ± 20 g was procured from the Experimental Animal Center of Wenzhou Medical University.

### 2.3. Equipment

Waters Xevo TQ-S Micro mass with ACQUITY H-Class UPLC (Waters Corp., Milford, MA, USA) was utilized in this study. Data acquisition and instrument control were performed using Masslynx 4.1 software (Waters Corp., Milford, MA, USA). Additionally, a Multifuge XIR low-temperature high-speed centrifuge (Hermos, Germany) was employed.

### 2.4. Chromatographic Conditions

The UPLC analysis was performed using a HSS T3 column (2.1 mm × 50 mm, 1.8 *μ*m) at a controlled temperature of 40°C. The mobile phase consisted of acetonitrile-0.1% formic acid in water, and the flow rate was maintained at 0.4 mL/min throughout the experiment. A total run time of 6.0 min was employed, following the gradient elution process outlined in Table 1.

### 2.5. Mass Spectrometry Conditions

Nitrogen was used as the desolventization gas (800 L/h) and cone gas (50 L/h). The capillary voltage was set at 2.2 kV, while the ion source temperature was maintained at 150°C. Desolventization was carried out at a temperature of 400°C using electrospray ionization (ESI) as the ion source.

**Figure 2:**
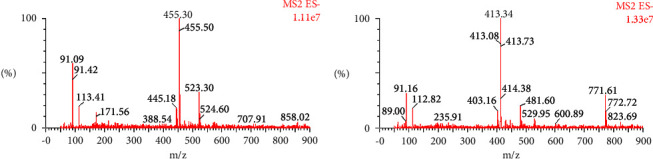
MS spectra of Rhodojaponin II and Rhodojaponin III.

**Figure 3:**
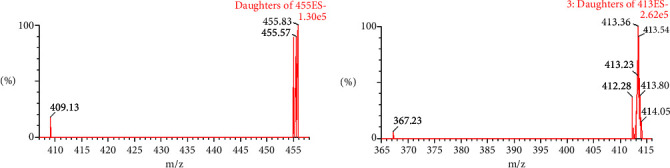
MS/MS spectra of Rhodojaponin II and Rhodojaponin III.

The daughter ions of Rhodojaponin II and Rhodojaponin III were not obvious (Figure 2), and then the mother ions were selected. Positive ion detection and multiple reaction monitoring (MRM) were employed for analysis, with Rhodojaponin II m/z 455.2 ⟶ 455.2 and Rhodojaponin III m/z 413.2 ⟶ 413.2 being the selected ions for quantitative analysis, Figure 3.

### 2.6. Sample Pretreatment

The plasma sample (50 *μ*L) was transferred into a 1.5 mL Eppendorf tube, followed by the addition of acetonitrile (200 *μ*L). After vortexing for 1.0 min, the mixture was centrifuged at 13,000 rpm for 10 min at 4°C. A volume of 2 *μ*L from the resulting supernatant was injected for subsequent analysis.

### 2.7. Preparation of Standard Curve

The Rhodojaponin II and Rhodojaponin III standards were accurately weighed, and a stock solution of Rhodojaponin II (0.5 mg/mL) and Rhodojaponin III (0.5 mg/mL) was prepared using methanol. Subsequently, the stock solution of Rhodojaponin II and Rhodojaponin III was diluted with acetonitrile to generate a series of standard working solutions. The plasma standard curves for Rhodojaponin II and Rhodojaponin III were established by combining blank rat plasma with an appropriate amount of standard working solution within the concentration range of 2–1250 ng/mL.

### 2.8. Method Validation

The UPLC-MS/MS method was validated according to the international regulatory guidelines (selectivity, lower limit of quantitation, accuracy, intraday/interday precision, matrix effect, recovery, and stability) [8, 9].

### 2.9. Pharmacokinetics

The pharmacokinetic study protocol was approved by the Laboratory Animal Ethics Committee of the First Affiliated Hospital of Wenzhou Medical University (WYYY-AEC-2023-144). Due to the inherent variability of SD rats, twelve rats were divided into two groups. The disparity in toxicity between Rhodojaponin II and Rhodojaponin III necessitates the need for refinement. Then, one group received intravenous injections of Rhodojaponin II at a dose of 0.5 mg/kg, while the other group received intravenous injections of Rhodojaponin III at a dose of 0.25 mg/kg. Blood samples (0.300 mL/rat/at one time point) were collected from the caudal vein at ten time points (0.0833 h, 0.5 h, 1 h, 2 h, 3 h, 4 h, 6 h, 8 h, 12 h, and 24 h). These samples were centrifuged at a rate of 3,000 revolutions per minute for ten minutes at −4°C; the obtained plasma samples were then stored at −20°C.

## 3. Results and Discussion

### 3.1. Selectivity

**Figure 4:**
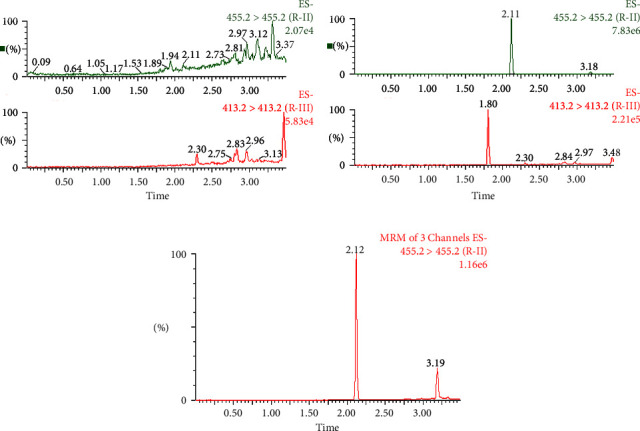
UPLC-MS/MS of Rhodojaponin II (*t*_*R*_ = 2.11 min) and Rhodojaponin III (*t*_*R*_ = 1.80 min) in rat plasma. (a) Blank rat plasma, (b) blank rat plasma spiked with Rhodojaponin II and Rhodojaponin III, and (c) a rat plasma sample after administration of Rhodojaponin II.

The blank plasma of rats was utilized and supplemented with Rhodojaponin II and Rhodojaponin III separately. Following the plasma treatment protocol outlined in Section 2.6, a specificity examination was conducted to obtain the corresponding chromatography, as depicted in Figure 4. No endogenous and cross-talk interferences was found in the retention time of Rhodojaponin II (*t*_*R*_ = 2.11 min) and Rhodojaponin III (*t*_*R*_ = 1.80 min).

### 3.2. Standard Curve and Lower Limit of Quantification

The solutions at concentrations of 2, 5, 12, 50, 125, 250, 500, and 1250 ng/mL were prepared by adding Rhodojaponin II and Rhodojaponin III to blank rat plasma (100 *μ*L) following the “sample handling” procedure. Subsequently, the samples were treated and their spectra recorded. Based on the calibration curves, the lowest concentration was identified as the lower limit of quantification (LLOQ). For Rhodojaponin II, the equation *y* = 4.8558*x* + 70.633 (*r* = 0.9991) was obtained, whereas for Rhodojaponin III it was *y* = 2.968*x* + 112.06 (*r* = 0.9991), where *x* represents the analyzed concentration and *y* denotes peak area. LLOQ of Rhodojaponin II and Rhodojaponin III was 2 ng/mL.

### 3.3. Accuracy, Precision, Matrix Effect, and Recovery

Four quality control (QC) samples with plasma concentrations of 2, 4, 100, and 1000 ng/mL were prepared for UPLC-MS/MS analysis following the same procedure as the standard curve preparation. Accuracy, precision, matrix effect, and recovery were assessed through six simultaneous preparations at each concentration. The precision for both intraday and interday measurements was below 15%, while the accuracy ranged from 88% to 115%.

Rat blank plasma was extracted and supplemented with Rhodojaponin II and Rhodojaponin III at concentrations of 2, 4, 100, and 1000 ng/mL (*n* = 6) in order to evaluate the matrix effect. The matching peak areas were then contrasted with those derived from pure standard solutions at comparable concentrations [10]. By comparing the peak area of extracted quality control samples with that of reference quality control solutions reconstituted in blank plasma extracts (*n* = 6), the recovery of both Rhodojaponin II and Rhodojaponin III was assessed. The matrix effect fell within the range of 90%–110%, and the recovery exceeded 78%. These values meet the criteria for drug analysis in biological samples outlined in Table 2.

### 3.4. Stability

The plasma samples (4, 100, and 1000 ng/mL) were subjected to three cycles of freezing and thawing after pretreatment, followed by incubation at room temperature for 24 hours. A stability test was conducted at −20°C for a duration of 30 days to assess their stability. Rhodojaponin II and Rhodojaponin III exhibited an accuracy range of 86–112%, with a within-group standard deviation of 13%. Based on these findings, both Rhodojaponin III and Rhodojaponin II demonstrated excellent stability.

The stability of the Rhodojaponin II (1000 ng/mL) and Rhodojaponin III (1000 ng/mL) standard solution tests was conducted at room temperature for 24 hours, and at −20°C for a duration of 30 days. It exhibited an accuracy range of 90–108%, with a standard deviation of 10%.

### 3.5. Pharmacokinetic Study

**Figure 5:**
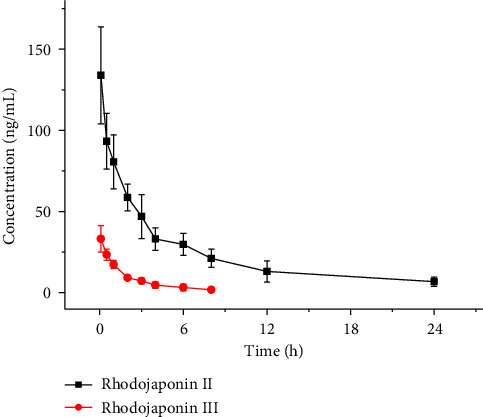
The concentration-time curve of rats after intravenous administration of Rhodojaponin II (iv, 0.5 mg/kg) and Rhodojaponin III (iv, 0.25 mg/kg).

Six rats were administered 0.5 mg/kg of Rhodojaponin II via the sublingual route, while another six rats received 0.25 mg/kg of Rhodojaponin III using the same administration method. The pharmacokinetic parameters were determined utilizing UPLC-MS/MS and analyzed employing Drug and Statistics (DAS) 2.0 software (Wenzhou Medical University, Wenzhou, China), as presented in Table 3, and concentration-time curve of rats is shown in Figure 5.

The concentration of Rhodojaponin III was determined using an UPLC-MS/MS method by Zhang et al., which was developed and validated over a range of 1–200 ng/mL. This method was then applied to investigate the pharmacokinetics of Rhodojaponin III in mice following intravenous (0.06 mg/kg) or oral (0.24 mg/kg) administration [3]. The results demonstrated rapid oral absorption of Rhodojaponin III with a time to peak concentration of 0.08 h, as well as favorable oral bioavailability (73.6%). Furthermore, both intravenous and oral administration resulted in quick elimination of Rhodojaponin III, with half-life values of 0.19 h and 0.76 h, respectively [3]. In contrast, the half-life (*t*_1/2_) of Rhodojaponin III after intravenous administration at a dose of 0.25 mg/kg was found to be 2.6 ± 1.3 h in this study.

Dong et al. developed a LC-MS method to detect the rat plasma concentrations of three major Rhodojaponins (Rhodojaponin II, and III) [11]. Notably, the pharmacokinetic parameters [(area under the plasma concentration-time curve (AUC), half-life (*t*_1/2_), time to reach maximum plasma concentration (*t*_max_), maximum plasma concentration (*C*_max_))] differed significantly among Rhodojaponin II, and III. After oral administration of *Rhododendri Mollis* Flos extract at doses of 21.44 mg/kg and 112.56 mg/kg respectively, the *t*_1/2_ values for Rhodojaponin II they were 133.74 ± 66.05 min and 215.96 ± 163.68 min; while for Rhodojaponin III they were 83.69 ± 39.57 min and 219.63 ± 91.11 min. While in this study, after intravenous administration, the *t*_1/2_ of Rhodojaponin II was determined to be 7.6 ± 4.3 h following intravenous administration, and the *t*_1/2_ of Rhodojaponin III was found to be 2.6 ± 1.3 h after intravenous administration, Table 3.

## 4. Conclusion

The UPLC-MS/MS method established for the determination of Rhodojaponin II and Rhodojaponin III in rats demonstrates desirable attributes, including specificity, accuracy, precision, matrix effect, and stability. These methods fulfill the methodological requirements and are suitable for high-throughput detection. This study showcases the potential of integrated pharmacokinetics and provides a valuable reference for a more comprehensive understanding of the pharmacokinetic behavior of *Rhododendron molle* (Blum) G. Don and its efficacy.

## Figures and Tables

**Table 1:** Structure of gradient elution mobile phases.

Runtime (min)	Acetonitrile (v/v%) (%)	0.1% formic acid in water (v/v%) (%)
0–0.2	10	90
0.2–2.4	10–75	90–25
2.4–5.0	75–90	25–10
5.0–5.1	90–10	10–90
5.1–6.0	10	90

**Table 2:** Accuracy, precision, matrix effect, and recovery of Rhodojaponin II and Rhodojaponin III in rat plasma (*n* = 6).

Compound	Concentration (ng/mL)	Accuracy (%)		Precision (RSD%)		Matrix effect (%)	Recovery (%)
		Intraday	Interday	Intraday	Interday		
Rhodojaponin II	2	114.8	88.4	13.1	13.4	102.0	83.0
	4	92.6	98.7	11.2	12.4	101.1	84.5
	100	106.6	96.7	9.4	4.5	106.2	86.6
	1000	97.2	101.3	6.5	9.0	109.2	85.4

Rhodojaponin III	2	95.5	97.2	12.7	14.7	90.5	81.7
	4	101.5	107.2	9.3	8.4	100.1	83.6
	100	100.1	109.2	7.8	10.3	106.8	78.8
	1000	103.1	103.9	6.8	6.1	99.7	85.9

**Table 3:** Main pharmacokinetic parameters after intravenous administration of Rhodojaponin II (iv, 0.5 mg/kg) and Rhodojaponin III (iv, 0.25 mg/kg) in rats (*n* = 6).

Compound	AUC_(0-t)_	AUC_(0-∞)_	*t*_1/2*z*_	CL_z_	*V*_*z*_	*C*_max_
	ng/mL *∗* h	ng/mL *∗* h	h	L/h/kg	L/kg	ng/mL
Rhodojaponin II	550.2 ± 97.6	631.0 ± 153.2	7.6 ± 4.3	0.8 ± 0.2	8.5 ± 4.1	133.9 ± 29.9
Rhodojaponin III	65.5 ± 12.1	74.3 ± 16.6	2.6 ± 1.3	3.5 ± 0.7	12.7 ± 4.0	33.2 ± 8.2

AUC: area under the plasma concentration-time curve; *t*_1/2_: half-life; CL: plasma clearance; *V*: apparent volume of distribution; *C*_max_: maximum plasma concentration.

## Data Availability

The data used to support the findings of this study are included within the article.
